# Effect of exposure to CeO_2_ nanoparticles on ram spermatozoa during storage at 4 °C for 96 hours

**DOI:** 10.1186/s12958-018-0339-9

**Published:** 2018-03-06

**Authors:** Laura Falchi, Grazia Galleri, Gian Mario Dore, Maria Teresa Zedda, Salvatore Pau, Luisa Bogliolo, Federica Ariu, Alessandra Pinna, Stefano Nieddu, Plinio Innocenzi, Sergio Ledda

**Affiliations:** 1 Dipartimento di Medicina Veterinaria, Università degli Studi di Sassari, Sassari, Italy; 2 Dipartimento di Medicina Clinica e Sperimentale, Università degli Studi di Sassari, Sassari, Italy; 3 Department of Materials, Imperial College London, South Kensington Campus, London, UK; 4 Dipartimento di Chimica e Farmacia, Università degli Studi di Sassari, Sassari, Italy

**Keywords:** Acrosome, CASA, DNA, Motility, Nanoparticles, Ovine, Oxidative stress, ROS, Spermatozoa

## Abstract

**Background:**

Cerium oxide nanoparticles (CeO_2_ NPs) are able to store and release oxygen, conferring them scavenger activity against oxidative stress. However, their effects in reproductive systems are not yet well understood. The aim of the study was to investigate the effects of exposure of refrigerated ram semen to CeO_2_ NPs for 96 h on the main structural and kinematic parameters of spermatozoa.

**Methods:**

The ejaculates of 5 Sarda rams were collected, pooled and diluted in a soybean lecithin extender. Samples were exposed to increasing doses of CeO_2_ NPs (0, 44 and 220 μg/mL) and stored at 4 °C for 96 h. Analyses of kinematic parameters (computer assisted sperm analysis, CASA), integrity of membranes (PI/PSA staining), ROS production (H_2_DCFDA staining) and DNA damage (sperm chromatin structure assay with acridine orange, SCSA) were performed every 24 h (0, 24, 48, 72 and 96 h of incubation). The experiment was carried out in 6 replicates. Data were analysed by repeated measures ANOVA with Bonferroni’s as post hoc test. When the assumption of normality was not met (ROS), non-parametric Kruskal-Wallis rank test was carried out.

**Results:**

Exposure of ram spermatozoa to increasing doses of CeO_2_ NPs had a beneficial effect on the main motility parameters from 48 h of incubation onward. Velocity of sperm cells was enhanced in the groups exposed to CeO_2_ NPs compared to the control. Incubation with NPs had beneficial effects on the integrity of plasma membranes of spermatozoa, with higher percentage of damaged cells in the control group compared to the exposed ones. Production of ROS was not affected by exposure to NPs and its levels rose at 96 h of incubation. The integrity of DNA remained stable throughout the 96 h of storage regardless of co-incubation with NPs.

**Conclusions:**

We reported beneficial effects of CeO_2_ NPs on kinematic and morphologic parameters of ram semen, such as motility and membrane integrity following 96 h of exposure. Furthermore, we also proved no genotoxic effects of CeO_2_ NPs. These effects could not be related to an antioxidant activity of CeO_2_ NPs, since ROS levels in exposed cells were similar to those of unexposed ones.

## Background

Over the past decades, nanoparticles of Cerium oxide (CeO_2_ NPs) have received considerable scientific interest due to their peculiar chemical, physical and biological properties. However, their everyday use in industry, food science and cosmetics is limited by the concerns about potential effects of their distribution and bioaccumulation in the environment [1]. More recently, the use of CeO_2_ NPs in biomedicine has been taken into account considering their capacity to store oxygen and consequent scavenger activity against reactive oxygen species (ROS) comparable to that of antioxidant enzymes in biological systems [2–4]. In literature, a large number of articles described a reduction in ROS levels in several tissues or cells following exposure to CeO_2_ NPs. Recently, scavenger action has been reported in cortical neurons in rats with spinal injuries [5], human keratinocytes [6], mice endothelial cells and fibroblasts [7], human breast and fibrosarcoma cells [8] and cardiac cells [9]. In contrast, many authors reported pro-oxidant effects especially in pulmonary cells [10, 11] and DNA damage in liver cells and leucocytes [12]. These diverging observations suggest that CeO_2_ NPs may show the same paradox activity described for other scavenger substances, perhaps depending on the physical and chemical characteristics of the compound, the concentration, the length of exposure and the biological system involved.

Few studies focussed on the effects of CeO_2_ NPs in the reproductive system and the findings are often in contrast. In mice, exposure of oocytes to increasing doses of CeO_2_ NPs led to oxidative stress and consequent DNA damage [13]. In the same species, a decrease in fertilization rates and accumulation in granulosa cells and sperm plasma membranes has been described [14]. However, in the ovine, gametes well tolerated co-incubation with CeO_2_ NPs. In particular, our research group reported that granulosa cells but not oocytes internalise this compound by endocytosis. Moreover, low concentrations of NPs enhanced in vitro fertilization of oocytes with low developmental competence possibly throughtheir scavenging action and downregulation of genes activated by oxidative stress [15].

Ram spermatozoa exposed to increasing concentrations of CeO_2_NPs for 24 h, showed no NPs uptake, occasionally sporadic contacts with plasma membranes and no adverse effects on DNA integrity and motility parameters. Furthermore, the redox balance of the cells was not perturbed by exposure to NPs since both ROS levels and mitochondrial activity remained stable [16]. We hypothesised that, submitting ram spermatozoa to a prolonged stressing condition such as extended storage at 4 °C and exposing them to CeO_2_ NPs would have triggered the scavenging action of this compound. Thus, the aim of the study was to investigate the effects of the exposure to increasing doses of CeO_2_ NPs on the kinematic parameters, integrity of membranes, DNA fragmentation and oxidative status of ram semen stored at 4 °C for 96 h.

## Methods

### Experimental design

**Fig. 1:**
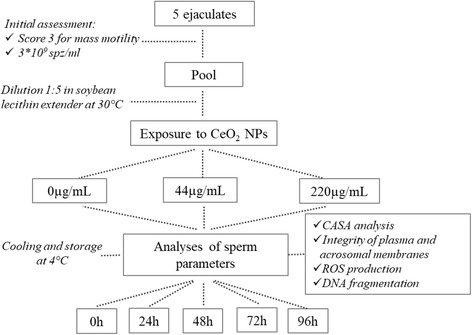
Experimental plan for the investigation of the effects of CeO_2_ NPs on ram semen stored at 4 °C for 96 h

The experimental design is described in Fig. 1. The ejaculates of 5 rams were collected and selected by mass motility [score ≥ 3 on a scale of 0–5 (0 = no motility, 5 = vigorous swirling waves of movements)], volume (≥ 0.5 mL) and sperm concentration (3x10^9^spz/mL). After selection, the samples were immediately pooled and diluted 1:5 (final concentration 600x10^6^spz/mL) in soybean lecithin extender OVIXcell (IMV Technologies) at 30 °C. The pool of ejaculates was divided in 3 aliquots that were supplemented with increasing doses of CeO_2_ NPs [0 (control), 44 and 220 μg/mL], gradually cooled to 4 °C in 2 h and stored at this temperature for 96 h. At different time points (0, 24, 48, 72 and 96 h), analyses on kinematic parameters, integrity of acrosome and plasma membranes, oxidative stress (ROS production) and DNA integrity were assessed in all 3 groups. The selection of the two doses of NPs was adopted from our previous study [16] and was close to the doses used in studies performed on somatic cells [7, 17] and gametes [15]. The experiment was carried out in 6 replicates.

### Animals and semen collection

Five rams of Sarda breed (2–3 years old) housed at the Genetic Centre of AGRIS (Agenzia Regionale per la Ricerca in Agricoltura, Bonassai, Italy) were selected for the present experiment. They were of proven fertility and their sanitary status was checked before starting the trial. Semen was collected by artificial vagina, placed in a 30 °C water bath and processed for initial evaluation (volume, concentration and mass motility) within 5 min.

### Cerium dioxide nanoparticles (CeO_2_ NPs)

Nanoparticles of CeO_2_ were synthesized according to the protocol reported by Falchi et al. [16].

### Motility analysis

Motility analysis was performed by computer assisted sperm analysis (CASA, Ivos, Hamilton Thorne, Biosciences). An aliquot of each sample was diluted in warm PBS (Dulbecco’s Phosphate Buffered Saline; 37 °C; 10–20 x 10^6^spz/mL), a 10 μL drop was placed on a warm slide (Leja slides, 20 μm, IMV Technologies, France) and loaded in the analyser. Six fields were selected and analysed in triplicate for: total motility (TM), progressive motility (PM), average path velocity (VAP), straight line velocity (VSL), curvilinear velocity (VCL), lateral head displacement (ALH), beat cross frequency (BCF), straightness (ratio VSL/VAP, STR), linearity (ratio VSL/VCL, LIN). Elongation (ELONG), area and velocity distribution (rapid, medium, slow and static spermatozoa) were also assessed.

### Plasma membrane integrity and acrosome status

Plasma membrane integrity and acrosome status were assessed by differential staining. An aliquot of semen (10 μL) from each group was added to 290 μL PBS (phosphate buffer saline), 4 μL PI (Propidium Iodide, 1 mg/mL; Sigma-Aldrich, USA) and 4 μL FITC-PSA (*Pisum sativum* agglutinin conjugated with fluorescein isothiocyanate; 1 mg/mL; Sigma-Aldrich, USA) and incubated in the dark for 15 min at 37 °C. Each sample was washed twice by centrifugation at 4229 RPM for 3 min. The final pellet was re-suspended in 250 μL PBS. A 10 μL drop was placed on a warm slide with a cover slip and observed under fluorescence microscope (Olympus IX70, Olympus Optical Co. Ltd, Japan). A total of 200 spermatozoa per slide were counted and classified in: viable spermatozoa with intact acrosome, PI-/PSA-; dead spermatozoa with intact acrosome, red, PI+/PSA-; viable spermatozoa with reacted acrosome, green, PI-/PSA+; dead spermatozoa with reacted acrosome, red and green, PI+/PSA+.

### Flow cytometer analyses

Flow cytometry was performed using the BD FACS Canto™ platform (BD Biosciences, USA) and the data were analysed by BD FACS DIVA software (BD Biosciences, USA). A total of 20,000 events per sample were acquired.

### ROS production

For the assessment of intracellular ROS production, sperm samples were stained with 2′,7’dichlorofluorescein diacetate (H_2_DCFDA, Sigma Aldrich, USA). Briefly, an aliquot (25 μL) of each sample was diluted in 1 mL PBS containing 10 μM H_2_DCFDA and incubated in the dark for 30 min at 38 °C. Following incubation, samples were centrifuged at 4229 RPM for 3 min, the supernatant was gently discarded, and the pellet was re-suspended in 500 μL of 2% paraformaldehyde and left at 4 °C for 1 h. After fixation, samples were centrifuged again at 4229 RPM for 3 min and supernatant was removed and replaced by 300 μL PBS. Samples were stored in the dark at 4 °C until flow cytometric analysis, which was performed within a month.

### Sperm chromatin structure assay

The integrity of DNA was assessed by SCSA. The labelling of fragmented DNA in fresh controls and in samples incubated with CeO_2_ NPs was carried out as previously described by Evenson et al. [18]. Briefly, an aliquot of 50 μL of each sample was diluted in 150 μL TNE buffer (0.15 M NaCl, 1 mM EDTA, 10 mM Tris, pH 7.2) at 4 °C, immediately plunged into liquid nitrogen and stored at − 80 °C until analysis. Frozen samples were thawed in crushed ice and 200 μL of sperm/TNE suspension was mixed to 400 μL of Acid Detergent Solution (0.08 M HCl, 0.15 M NaCl, 0.1% Triton X-100, pH 1.4). After 30 s, 1.2 mL of staining solution (0.15 M NaCl, 1 mM EDTA, 10 mM Tris, 0.2 M NA_2_HPO_4_, 0.1 M citric acid, pH 6) containing 6 μg/mL of Acridine Orange, was added to the mixture. Within 3 min from the staining, the samples were analysed by the flow cytometer. The levels of DNA fragmentation were assessed calculating the DFI (DNA fragmentation index) as the ratio of red fluorescence and total fluorescence (green + red).

### Statistical analysis

Statistical analysis was performed using Stata 11.2/IC (StataCorp LP, USA). Normal distribution of data was checked by Shapiro-Wilk test. When the assumption was met, data were analysed by Analysis of Variance with repeated measures with Bonferroni’s as post hoc test. When the assumption of normality was not met (ROS), non-parametric Kruskal-Wallis rank test was performed.

## Results

### Kinematic parameters assessed by CASA

Motility parameters are important predictors of male fertility since they indicate the ability to move into the female genital tract to reach the fertilization site. To assess the potential effects of the exposure of ram spermatozoa to CeO_2_ NPs on kinematic parameters, CASA analysis was performed every 24 h for 96 h. The results showed that the exposure to increasing doses of CeO_2_ NPs had a significant effect on the main kinematic parameters from 48 to 72 h onward whereas the time of incubation affected all of them (*P* < 0.05).

**Fig. 2:**
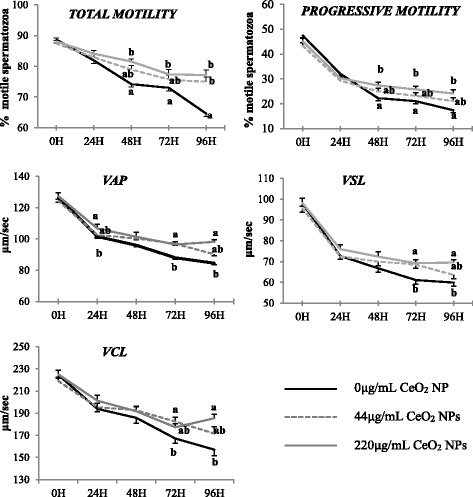
Main kinematic parameters assessed by CASA of ram spermatozoa exposed to CeO_2_ NPs for 96 h at 4 °C. Different letters (^a, b^) indicate significant differences among treatments within time point (*P* < 0.05). Results are shown as means ± SEM

In detail, most of the main kinematic parameters (PM, VAP, VSL, VCL) showed a sharp decrease in the first 24 h of storage at 4 °C independently of the exposure to CeO_2_ NPs. The decline was steeper for PM (around 15% drop) and for VSL (around 30% drop). Total motility had a gradual time-dependent decrease although from 48 h onward sperm cells incubated with 220 μg/mL CeO_2_ NPs showed a significantly higher TM and PM compared to the control group (*P* < 0.05). A similar pattern was observed for VAP, VSL and VCL, but the effect was significant from 72 h of incubation onward (*P* < 0.05; Fig. 2). Sperm cells incubated with 44 μg/mL NPs showed an intermediate pattern, with no significant differences from both control and 220 μg/mL groups (*P* > 0.05).

**Fig. 3:**
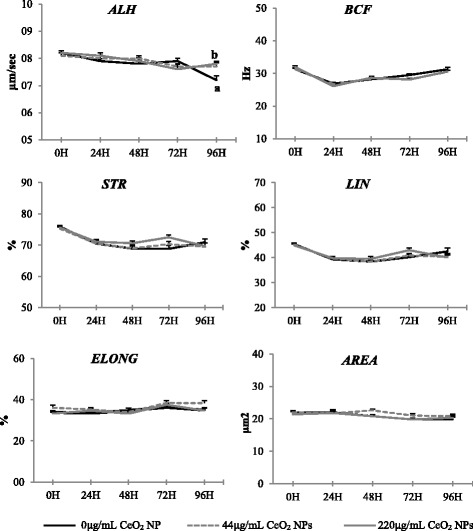
Secondary kinematic parameters assessed by CASA of ram spermatozoa exposed to CeO_2_ NPs for 96 h at 4 °C. Different letters (^a, b^) indicate significant differences among treatments within time points (*P* < 0.05). Time had a significant effect on the analysed parameters (*P* < 0.05). Results are shown as means ± SEM

As described in Fig. 3, the incubation with NPs had no influence on most of the secondary kinematic parameters (BCF, STR, LIN, ELONG and AREA) at any time point of the experiment (*P* > 0.05). Lateral head amplitude (ALH) was the only secondary parameter significantly affected by exposure to CeO_2_ NPs (*P* < 0.05). However, the effect was visible only at 96 h of incubation, when the control group showed significantly lower ALH compared to exposed groups (*P* < 0.05). The time of exposure significantly affected all secondary parameters (*P* < 0.05) except for ELONG and AREA, that remained stable throughout the experiment.

**Fig. 4:**
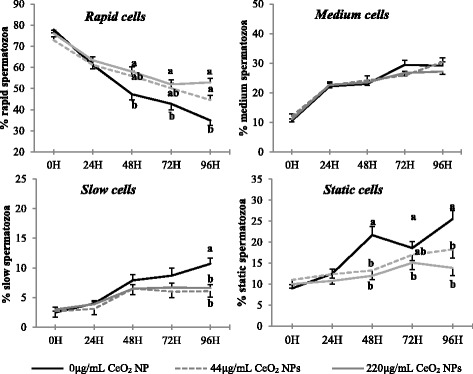
Velocity distribution of ram spermatozoa exposed to increasing doses of CeO_2_ NPs and stored for 96 h at 4 °C. Different letters (^a, b^) indicate significant differences among treatments within time points (*P* < 0.05). Results are shown as means ± SEM

Concerning the analysis of velocity distribution (Fig. 4), the collected data showed a pattern similar to that of TM and PM. In detail, CeO_2_ NPs had no influence on velocity distribution during the first 24 h of exposure but, from 48 h onward, sperm cells incubated with 220 μg/mL NPs were consistently more rapid and less static compared to the control group (*P* < 0.05); sperm cells exposed to 44 μg/mL NPs showed an intermediate pattern. No effect of CeO_2_ NPs was observed on the percentage of medium cells at any time point of the experiment (*P* < 0.05). The percentage of slow cells gradually increased in all groups in the first 24 h remaining stable in exposed sperm cells from the 48 h onward. Conversely, in control sperm cells, it continued to increase until 96 h showing significantly higher rates compared to exposed groups (*P* < 0.05).

### Integrity of plasma membrane and acrosome

**Fig. 5:**
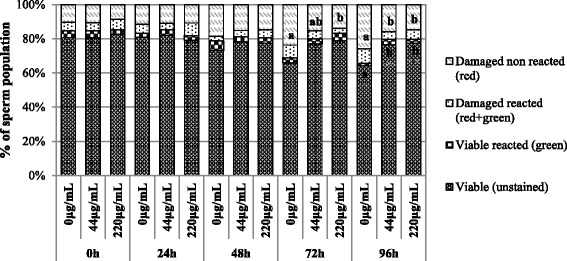
Integrity of cytoplasmic and acrosomal membranes of spermatozoa incubated with CeO_2_ NPs for 96 h at 4 °C. Different superscripts (^a, b^) indicate significant differences for *P* < 0.05 among treatments within time points

In the ram, plasma and acrosomal membranes of sperm cells, that play an important role during sperm capacitation and fertilization, can be easily damaged during storage procedures leading to decrease in semen quality and consequently in fertilizing ability. To test the effects of the exposure to CeO_2_ NPs on plasma and acrosomal membranes, the differential staining with PI/PSA was used. As described in Fig. 5, incubation time and CeO_2_ NPs exposure had an overall significant effect on the percentage of viable (unstained, PI-/PSA-) sperms and of sperms with damaged plasma membranes (PI+/PSA-; *P* < 0.05). In general, no effect of co-incubation with CeO_2_ NPs was observed in the first 48 h of exposure, during which the rates of viable and damaged spermatozoa remained stable. However, at 72 h and 96 h, the 220 μg/mL group had a higher percentage of viable spermatozoa compared to the control (*P* < 0.05); whereas at 96 h a significant difference was also found between the 44 μg/mL and the control group (*P* < 0.05). Conversely, at 72 h and 96 h the percentage of damaged non reacted spermatozoa was significantly higher in the control compared to the 220 μg/mL group (*P* < 0.05). No time and treatment effects were observed in the percentages of reacted (PI-/PSA+) and dead reacted (PI+/PSA+) spermatozoa among groups at any time point of the experiment (*P* > 0.05; Fig. 5).

### Production of ROS

**Fig. 6:**
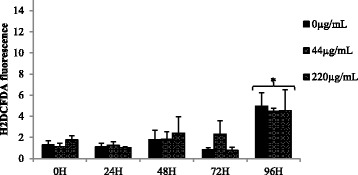
Reactive oxygen species (ROS) production by ram semen exposed to increasing concentrations of CeO_2_ NPs. Results are shown as means ± SEM of fluorescence following H_2_DCFDA staining. Asterisk indicates significant difference (*P* < 0.05) between time points

Since ram spermatozoa are susceptible to oxidative stress with overproduction of ROS during storage at low temperatures, we tested the effects of CeO_2_ NPs on ROS levels in sperm cells stored for 96 h at 4 °C. The results, represented in Fig. 6, showed that increasing doses of CeO_2_ NPs did not affect the production of ROS at any time point of the experiment (*P* > 0.05). Conversely, time of storage had a significant effect on the oxidative status of sperm cells stored for 96 h. In detail, ROS levels remained fairly stable for 72 h with no differences among groups, while the levels rose abruptly at 96 h with a 3- to 4-fold increase (*P* < 0.05; Fig. 6).

### DNA fragmentation

**Fig. 7:**
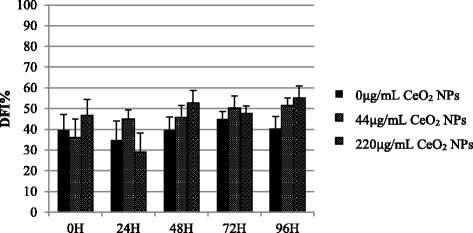
Sperm chromatin structure assay in ram spermatozoa incubated with increasing concentrations of CeO_2_ NPs for 96 h. The DFI% was calculated as the ratio red fluorescent cells (single strand DNA)/total fluorescent cells [red + green (double strand DNA)]. Results are shown as mean percentages ± SEM

Since there is no consensus on the effects of CeO_2_ NPs on DNA integrity of somatic cells or gametes, we measured the levels of DNA fragmentation through SCSA in ram sperm cells exposed to NPs for 96 h. The results showed that DNA of sperm cells exposed to NPs well tolerated co-incubation with these compounds. No significant differences in DFI% were found among treated and control groups at any time point of the experimental trial (*P* > 0.05). Moreover, time effect was also not significant (*P* > 0.05; Fig. 7).

## Discussion

In this study, we reported the effects of exposure to CeO_2_ NPs of ram spermatozoa stored for 96 h at 4 °C. The choice of these experimental conditions was based on the hypothesis that CeO_2_ NPs, with their catalytic action, might prevent the deleterious effects of storage at low temperatures on ram sperm cells. The decrease in quality of refrigerated semen restricts its application during artificial insemination programs to a short time span, limiting the diffusion of superior genotypes to small geographic areas and to restricted numbers of selected animals. For this reason, the perspective of storing semen for longer times would represent a great benefit in the management of genetic selection and reproduction in sheep breeding systems and a valuable alternative to cryopreserved semen.

Over the past decades many research groups focussed on improving the quality of refrigerated ram semen. The most promising approach is represented by the supplementation of storage extenders with anti-oxidant substances that contrast the deleterious effects of ROS accumulation and oxidative stress on sperm cells. Among others, CeO_2_ NPs are gaining increasing interest for their ability to change their oxidation status [19] conferring them promising scavenger properties.

Our research group [16] previously reported that ram spermatozoa stored at 4 °C for 24 h well tolerated concentrations of CeO_2_ NPs above those commonly dispersed in the environment (water 0.024 mg/L [20]; soil 1.12 mg/kg [21]) and the results reported in the present study are in agreement with these observation. The potential toxic effects of these compounds have to be carefully assessed and the consequences on the reproductive system are still under investigation. In mice, low concentrations of CeO_2_ NPs significantly affected in vitro fertilization and had genotoxic effects on both male and female gametes [13, 14]. In the present study, we can state that none of the sperm parameters analysed was negatively affected by extended exposure of sperm cells to CeO_2_ NPs.

Moreover, we described a consistent and significant increase in kinematic parameters of spermatozoa incubated with high concentrations of NPs that has not been reported before. This beneficial effect was mostly evident in spermatozoa exposed to 220 μg/mL NPs from 48 h of incubation onward. In fact, in the first 24 h of the trial, CeO_2_ NPs did not exert any effect on any of the analysed parameters. It has been reported that the overall quality of ram semen stored at 4 °C dramatically drops after 3 days [22–24] and we can speculate that the activity of NPs might be stronger in cells with compromised morphologic and structural characteristics under stressing conditions such as storage at low temperatures.

In addition to the enhancement of TM and PM, incubation with NPs promoted the increase in parameters related to cells velocity (VAP, VCL and VSL) of around 10 μm/s. It has been reported that these parameters are highly correlated to fertility in several species such as ram [25, 26], bull [27] and boar [28]. Incubation with NPs also changed the rates of rapid spermatozoa in the exposed groups compared to the control with more than 50% of cells having a VAP > 75 μm/s at 72 and 96 h of incubation. These results are in agreement with what previously reported in rats fed with a diet containing citrate-coated CeO_2_ NPs (1 mg/kg). After 10 days of diet, the authors reported a significant increase in motility and viability of epididymal spermatozoa in treated animals compared to control group and suggested NPs as a helpful tool in contrasting age-related infertility [29]. A recent study carried out on humans reported an improvement in motility parameters in frozen- thawed semen following supplementation of cryopreservation medium with ZnO NPs [30]. These promising results may pave the way for a use of NPs as preservers of semen quality during storage at low temperatures although further investigations are strictly needed. Preaubert et al. reported no effect of CeO_2_ NPs on progressive motility rates in mice spermatozoa [14] and detrimental effects of NPs on sperm motility have been described in bull [31] and human [32], suggesting species-specific effects of these compounds.

Data on motility correlated well with the results obtained by the analysis of the integrity of plasma and acrosomal membranes suggesting a high biocompatibility of CeO_2_ NPs. As for kinematic parameters, NPs did not influence the status of the membranes in the first 48 h of exposure, but from 72 h onward, the effects of co-incubation were visible on spermatozoa exposed to NPs, that preserved more efficiently the morphologic structure of plasma membranes compared to unexposed cells. Acrosomes were not affected by exposure to NPs and this suggests a differential sensitivity of sperm membranes to stressing conditions. The absence of membrane alterations during incubation with NPs has been previously observed in the same species [16]. Moreover, no interaction with membranes or up-take but only occasional contacts between NPs and the post-acrosomal region of spermatozoa was described [16], suggesting that the positive effects triggered by NPs on sperm parameters are not mediated by intra-cellular mechanisms.

In this experiment, we exposed spermatozoa to a prolonged stressing factor (storage at 4 °C for 96 h) and investigated the role of CeO_2_ NPs in preventing oxidative sperm damage. However, the levels of ROS remained fairly stable until 72 h and then rose at 96 h in exposed and unexposed cells.

During storage procedures at low temperatures, ram spermatozoa are extremely sensitive to oxidative stress due to the high ratio unsaturated/saturated fatty acids in phospholipids of plasma membranes [33]. Disruption of intra-cellular balance of free radicals accumulation leads to impairment of quality parameters such as viability, motility, membranes integrity and consequent fertilizing ability, as reviewed by Bansal and Bilaspuri [34]. On the other side, ROS play a critical role in several essential physiological processes such as sperm binding, capacitation and hyper-activation, as recently reviewed by O’Flaherty et al. [35].

Although we reported a positive effect of CeO_2_ NPs in preserving quality traits in semen from 48 h onward, we could not correlate this effect with changes in the levels of ROS. This controversial result suggests that NPs, in the specific experimental conditions of the present trial, acted through pathways that are independent from ROS accumulation in sperm cells.

We could also speculate that CeO_2_ NPs might exert their catalytic action in the extra-cellular compound (storage extender) perhaps through their SOD or catalase mimetic behaviour, rather than inside sperm cells. This hypothesis is supported by the previously reported lack of uptake and internalisation of CeO_2_ NPs by sperm cells [16]. These external actions possibly depend on chemical and physical characteristics of NPs and interaction with the compounds involved in storing spermatozoa, such as extender and seminal plasma. In physiological conditions, SOD and catalase antioxidant activities have been detected in seminal plasma of several species like bull [36], boar [37] and equine [38], where they are involved in maintaining integrity of membranes and sperm function. We can also suggest that supplementation of storage extender with CeO_2_ NPs might have supported the activity of the enzymatic systems formerly present in seminal plasma of rams.

Perrin et al. reported that exposure of human spermatozoa to CeO_2_ NPs has genotoxic effects that were limited by the use of an antioxidant (L-Ergothioneine) suggesting a pro-oxidant activity of the NPs [39]. In our experiment, we did not report any genotoxic effect of CeO_2_ NPs at any time of the experimental trial. The levels of DNA fragmentation remained stable for up to 96 h and no differences were found with unexposed spermatozoa. This indicates that the biocompatibility of these compounds may depend by several factors; among others, a species sensitivity should not be excluded. In the mouse, CeO_2_ NPs induce DNA damage in both oocytes and spermatozoa affecting in vitro fertilization [14] whereas in the ovine species, supplementation of maturation media with CeO_2_ NPs (44μg/mL) did not impair but enhanced fertilization and blastocyst rate and no adverse effects were observed in chromatin configuration of oocytes exposed to NPs [15]. On the other side chromatin damage has been reported in mice [40] and bull spermatozoa [41] exposed to silver NPs.

## Conclusions

To our knowledge, this is the first report describing beneficial effects of CeO_2_ NPs on morphologic and kinematic parameters of ram semen, such as motility and plasma membrane integrity after 96 h of exposure. We also reported no genotoxic effects of these NPs. However, these beneficial effects could not be explained by an intra-cellular antioxidant activity exerted by these compounds, since ROS levels in exposed cells were similar to those of unexposed ones. Eventually, further investigations are needed to support these preliminary results and to pave the way to future applications of these compounds in reproductive biology.

## Abbreviations

ALH: Lateral head displacement
BCF: Beat cross frequency
CASA: Computer assisted sperm analysis
DFI: DNA fragmentation index
ELONG: Elongation
H_2_DCFDA: Dichlorofluorescein diacetate
LIN: Linearity
NPs: Nanoparticles
PBS: Phosphate buffer saline
PI: Propidium Iodide
PM: Progressive motility
PSA: *Pisum sativum* agglutinin
ROS: Reactive oxygen species
RPM: Revolutions per minute
SCSA: Sperm chromatin structure assay
STR: Straightness
TM: Total motility
TNE: Tris-NaCl-EDTA
VAP: Average path velocity
VCL: Curvilinear velocity
VSL: Straight line velocity
